# Long-term renal survival of γ3-heavy chain deposition disease: a case report

**DOI:** 10.1186/s12882-017-0645-z

**Published:** 2017-07-17

**Authors:** Takayuki Katsuno, Shige Mizuno, Masatsuna Mabuchi, Naotake Tsuboi, Atsushi Komatsuda, Shoichi Maruyama

**Affiliations:** 1 Department of Nephrology, Nagoya University Graduate School of Medicine, 65 Tsurumai-cho, Showa-ku, Nagoya, 466-8550 Japan; 2 Department of Internal Medicine, Kaikoukai Jyousai Hospital, Nagoya, Japan; 3 Department of Nephrology, Konan Kosei Hospital, Konan, Japan; 4 Department of Hematology, Nephrology, Rheumatology, Akita University Graduate School of Medicine, Akita, Japan

**Keywords:** Heavy chain deposition disease, Nephrotic syndrome, Chemotherapy, Melphalan, Monoclonal gammopathy of renal significance, Case report

## Abstract

**Background:**

Monoclonal immunoglobulin deposition disease (MIDD) is characterized by the non-amyloid deposition of monoclonal immunoglobulin fragments in the basement membranes. Heavy chain deposition disease (HCDD) is a type of MIDD. HCDD is an extremely rare disease, and only three cases have been reported in Japan up to the present. The prognosis of HCDD is very poor, and optimal treatment has not been established. Only a few cases of HCDD with favorable long-term renal prognosis have been reported to date.

**Case presentation:**

The authors describe a 61-year-old woman who presented with massive proteinuria, progressive kidney impairment, and hypocomplementemia. Kidney biopsy was performed for a precise diagnosis. On light microscopy, glomerules were lobulated and presented with nodular sclerosing glomerulopathy with membranoproliferative glomerulonephritis-like features. Immunofluorescence studies were positive for IgG, C3, and C1q within the mesangial nodules and in a linear distribution along the capillary walls without associated deposition of light chains. Staining for IgG showed the presence of linear deposits along tubular basement membranes. The analysis of the IgG subclass stain demonstrated intense positivity for IgG3 only. Electron microscopy revealed non-organized electron-dense deposits in the expanded mesangial area and inner aspect of the glomerular basement membranes. In accordance with the histological findings, we diagnosed γ3-HCDD. There was no evidence of plasma cell dyscrasia as a result of bone marrow aspiration. Serum and urine monoclonal proteins were not detected by immunoelectrophoresis and immunofixation electrophoresis. The serum free light chain ratio was within normal range. At first, prednisolone was administrated at a dose of 40 mg/day. However, a therapeutic effect was not observed. Urinary protein was not decreased and renal function further deteriorated. Therefore, melphalan plus prednisolone (MP) therapy was initiated. After 4 courses of MP therapy, the clinical parameters, including proteinuria, serum creatinine, albumin, and complement level (C3 and C4) were ameliorated. To date, the patient has been followed for 28 months, and long-term renal survival has been observed.

**Conclusions:**

In this case, hematologic disease such as multiple myeloma was not detected; however, MP therapy was effective. Recently, the novel concept of monoclonal gammopathy of renal significance (MGRS) has been reported. MIDD, which includes HCDD, is one category of MGRS. In MGRS, aggressive chemotherapy may induce favorable renal outcomes.

## Background

Monoclonal immunoglobulin deposition disease (MIDD) has three distinct forms: light chain deposition disease (LCDD), light and heavy chain deposition disease (LHCDD), and heavy chain deposition disease (HCDD). These three subtypes are distinguished depending on the components of the deposits. The deposits are derived from monoclonal light chains only in LCDD, monoclonal light and heavy chains in LHCDD, and monoclonal heavy chains only in HCDD. Renal involvement is frequent in MIDD. The main clinical features are renal dysfunction and proteinuria, often accompanied by nephrotic syndrome (NS). The characteristic pathological findings in MIDD are the presence of linear non-organized monoclonal immunoglobulin deposits along tubular basement membranes (TBM) and glomerular basement membranes (GBM) by immunofluorescence and electron microscopy. Especially in cases of HCDD, light microscopy shows nodular sclerosing glomerulopathy. Among cases of MIDD, HCDD is extremely rare. This rare disease was first described in 1992 [1]. To date, more than 20 years have elapsed from the first publication, only a small number of articles have been presented, and most of them are case reports [1–14]. In Japan, only three cases have been reported until now [2, 5, 11]. HCDD is classed into three subtypes (γ, α, and μ) depending on the components of the heavy chain deposits. IgG has four subclasses, and γ-HCDD is further classified into four subtypes (γ1–4). As a treatment option for MIDD including HCDD, effectiveness such as blood stem-cell autografting, alkylating agents, rituximab and thalidomide has been reported in the previous reports [7, 14–16]. However, the optimal therapeutic method for MIDD remains clearly undefined, and many cases have shown poor outcomes [3, 8, 9, 12–14, 17]. In HCDD, deletion of the first constant heavy chain domain (CH1) might be one possible pathogenesis [9, 14, 17, 18]. Deletion of the CH1 is a required condition for the secretion of a free monoclonal heavy chain by the underlying clonal plasma cell disorder [19]. Based on this pathogenesis, therapies targeting pathological plasma cell clones may be useful. Soma et al. and Oe et al. reported two cases of HCDD treated successfully using melphalan and prednisolone (MP) therapy [2, 5]. Several reports have shown beneficial results with high-dose melphalan followed by autologous stem cell transplantation [20–23]. We herein describe a case of γ3-HCDD which demonstrated long-term renal survival after MP therapy. This is the fourth case in Japan.

## Case presentation

A 61-year-old woman with a medical history of hypertension and chronic kidney disease (CKD) was admitted to our hospital for proteinuria and progressive deterioration of renal function. She attended the hospital as an outpatient and was administered olmesartan, cilnidipine, and azosemide. Her serum creatinine level was 1.0 mg/dL, and no urinary abnormalities were observed in the local clinic. 2 months before admission, there were subjective symptoms of pedal edema and blood pressure elevation. Her creatinine level increased to 1.52 mg/dL, and heavy proteinuria was observed at a local clinic. Subsequently, she was referred to our hospital for further management.

At the initial visit, the patient had a blood pressure of 135/88 mmHg, body height of 149.5 cm, and weight of 42.5 kg. Physical examination revealed bilateral edema of the lower extremities. Urinalysis showed proteinuria with protein excretion of 4.15 g/g creatinine and 5–8 red blood cells per high-power field. Hematologic tests indicated a white blood cell count of 6.3 × 10^3^/μL, hemoglobin level of 12.0 g/dL, and platelet count of 315 × 10^3^/μL. Laboratory investigation revealed a serum creatinine concentration of 1.85 mg/dL, blood urea nitrogen of 37 mg/dL, and estimated glomerular filtration rate of 22.8 mL/min/1.73 m^2^. The total serum protein level was 5.2 g/dL with a serum albumin level of 3.1 g/dL. The following laboratory data were obtained: total cholesterol: 280 mg/dL, low-density lipoprotein cholesterol: 175 mg/dL, triglycerides: 237 mg/dL, uric acid: 6.3 mg/dL, C-reactive protein: 0.03 mg/dL. Liver dysfunction, diabetes mellitus, and electrolyte imbalance were not observed. Immunological studies showed a serum IgG concentration of 283 mg/dL (normal range 870–1700 mg/dL), IgA of 259 mg/L (normal range 110–410 mg/dL), and IgM of 183 mg/dL (normal range 35–220 mg/dL). The serum complement C3 level was 33.2 mg/dL (normal range 86–160 mg/dL), C4 was 3.3 mg/dL (normal range 17-45 mg/dL), and total hemolytic activity (CH50) was 9.1 U/mL (normal range 31.6–57.6 U/mL). Marked hypocomplementemia was recognized. Tests for hepatitis B and C, rheumatoid factor, anti-nuclear antibody, anti-DNA antibody, anti-SS-A/SS-B antibody, anti-neutrophil cytoplasmic antibody, anti-cardiolipin antibody, and cryoglobulin were all negative.

She was admitted to our hospital 2 days after the initial consultation for a careful examination. As a result of the additional examinations, the serum and urine immunoelectrophoresis showed no monoclonal bands. Furthermore, the entity of monoclonal protein was not identified on immunofixation electrophoresis. The serum free κ light chain level was 45.1 mg/L, λ light chain was 57.6 mg/L, and κ/λ ratio was 0.783 (normal range 0.248–1.804). Serum amyloid A protein level was not elevated at 2.7 μg/mL (normal 0–8.0 μg/mL). In the bone marrow examination, the CD 138 positive cells proliferate slightly, but they were not clear tumorous proliferation. Plasma cells are 0.5% in the bone marrow and heteromorphy has not been confirmed. Pathological proliferation of plasma cells was also denied by flow cytometry. Ultrasound examination indicated renal atrophy; therefore, laparoscopic renal biopsy was performed for accurate diagnosis.

### Renal biopsy

The kidney specimens were studied by light microscopy, immunofluorescence stain, and electron microscopy using standard techniques. For direct immunofluorescence stain, 3-μm cryostat sections were stained using polyclonal fluorescein isothiocyanate (FITC)-conjugated antibodies to IgG, IgA, IgM (Medical & Biological Laboratories, Nagoya, Japan), C3, C4, C1q, κ, and λ(Dako, Copenhagen, Denmark). Further staining of the IgG subclasses was performed using mouse monoclonal antibodies to human IgG1 (Bio-Rad Laboratories, California, USA), IgG2, IgG3 (Sigma-Aldrich, St. Louis, USA), and IgG4 (Binding Site, Birmingham, UK), followed by FITC-conjugated goat anti-mouse IgG (Jackson Immuno Research Inc., Pennsylvania, USA).

**Fig. 1:**
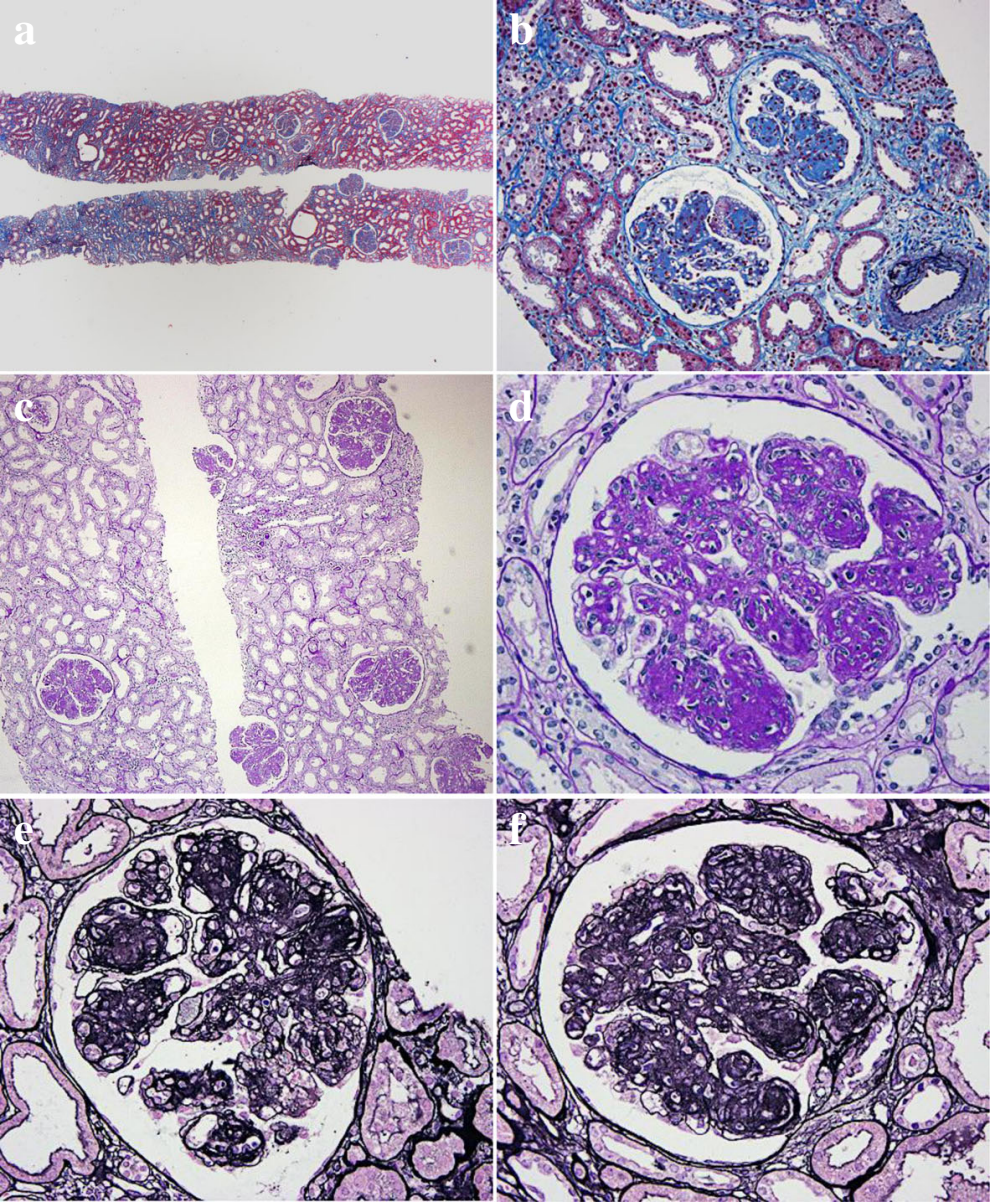
**a** and **b** On light microscopy, 13 glomerules are present, and none are globally sclerotic. All glomerules reveal similar findings. The interstitial architecture shows mild interstitial fibrosis and tubular atrophy. There is focal infiltration of inflammatory cells. Cast formation is not observed. Masson’s trichrome staining, original magnification (**a**) 40 (**b**) 200×. (**c**) and (**d**) Representative glomerules are lobulated and have nodular mesangial sclerosis with mesangial cell proliferation. Periodic acid-Schiff staining, original magnification (**c**) 100× (**d**) 400×. (**e**) and (**f**) Representative glomerules show nodular glomerulosclerosis markedly. Capillary walls are thickened, and duplication of glomerular basement membranes is observed. Endocapillary proliferation with mesangiolysis is detected in the focal segmental lesion. Adhesion to Bowman’s capsule is also seen. Periodic acid-methenamine silver staining, original magnification 400×

**Fig. 2:**
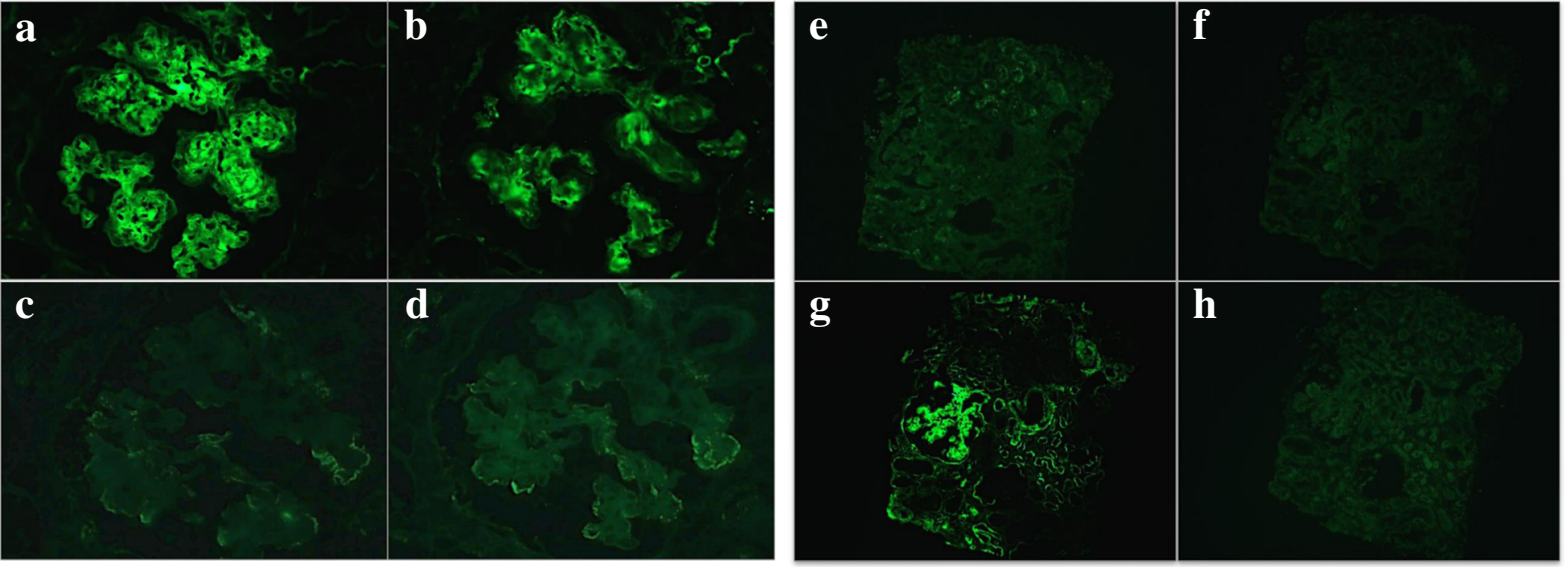
Immunofluorescence staining shows moderate positivity for IgG (**a**) and C3 (**b**) within the mesangial nodules and in a linear deposition along the capillary walls. However, κ (**c**) and λ (**d**) are undetectable in the same glomerule. Original magnification 400×. **e-h** Immunofluorescence findings for IgG subclasses. Only IgG3 (**g**) is intensely positive in the mesangium area and along the glomerular and tubular basement membranes. Staining for IgG1 (**e**), IgG2 (**f**), and IgG4 (**h**) was absolutely negative in the same specimen. Original magnification 100×

**Fig. 3:**
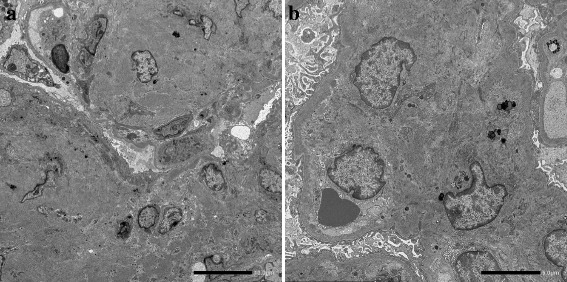
**a** and **b** Electron microscopy. Non-organized deposits are observed along the inner aspect of the glomerular basement membranes. Large size electron-dense deposits without organized structures are located in the expanded mesangium

The sample contained 13 glomerules, none of which were globally sclerosed. By light microscopy, glomerules showed a diffuse increase in the mesangial matrix and mild mesangial hypercellularity. Most glomeruli were lobulated and exhibited remarkable nodular formation in the mesangial area that narrowed the capillary lumens. Capillary walls were irregularly thickened. Duplication of the GBM accompanied by endothelial cell swelling indicated an endothelium disorder. Endocapillary proliferation and mesangiolysis were observed in several glomeruli. Crescents were not detected in the specimen. Tubular atrophy and interstitial fibrosis was mild with patchy infiltration of mononuclear cells. Vessels showed moderate arteriosclerosis (Fig. 1). Congo red staining was negative. Immunofluorescence staining showed moderate-intensity (2+) for IgG and C3 and mild intensity (1+) for C1q in the mesangium and along the capillary walls. Staining for IgG also was positive on TBM. However, no staining for light chains was observed in the mesangium, along the capillary walls, or on the TBM. IgG subclasses showed intense staining for IgG3, but IgG1, IgG2, and IgG4 were entirely negative (Fig. 2). Electron microscopy revealed electron-dense deposits (EDD) without organized structures along the inner aspect of the GBM. In the expanded mesangium area, nodular EDD were observed (Fig. 3). The pathological findings in this case corresponded with γ3-HCDD.

### Clinical course

Initially, the patient was treated with oral prednisolone (PSL) at a dose of 40 mg/day. However, the therapeutic response was very poor. The serum creatinine level increased to 3.65 mg/dL and blood urea nitrogen to 122 mg/dL, while the severe hypoalbuminemia (2.4 g/dL) did not improve. Urinary protein excretion did not decrease (4.27 g/g creatinine on admission to a maximum of 12.02 g/g creatinine), and in addition, the ascitic fluid and bilateral edema of the lower extremities became worse. The serum C3, C4, and CH50 titers remained extremely low (C3 19.7 mg/dL, C4 7.3 mg/dL, and CH50 13.6 U/mL). Therefore, the patient was treated with chemotherapy consisting of melphalan and PSL at a dose of 40 mg/day for 4 consecutive days. The daily use of PSL was rapidly tapered and stopped after chemotherapy. MP therapy was conducted 4 times in 7 months. The treatment interval was 1 month at short and 3 months at long. The dosage of melphalan was adjusted in accordance with renal function, 4 mg in the first and second courses, 6 mg in the third and 8 mg in the final. After 4 courses of MP therapy, the serum C3 and C4 titers were markedly elevated (C3 84.6 mg/dL and C4 31.3 mg/dL), and the serum creatinine level was decreased to 1.52 mg/dL. After discontinuance of MP therapy, the laboratory data did not deteriorate. To date, the patient has been followed for 28 months. The serum albumin levels have been maintained at over 3 g/dL, and the quantitative analysis of urinary protein was decreased to 2.72 g/g creatinine. NS was not observed and renal function was stable (the serum creatinine level was around 2.0 mg/dL). The patient’s condition improved with no edema, which had been observed initially.

## Discussion

We report a case of γ-3HCDD which was diagnosed with clinicopathological features. Written informed consent was obtained from the patient for the administration of melphalan. She provided informed consent for the publication of this case report.

Kambham et al. described the disease spectrum of HCDD [17]. The most common clinical findings are NS, hypertension, microhematuria, and, in some cases, hypocomplementemia. Progressive renal dysfunction is usually present at the time of diagnosis. Lin et al. reported the clinicopathologic findings and outcomes of MIDD [18]. Of the 34 patients, 6 patients were diagnosed with HCDD. At baseline, the mean serum creatinine level was 4.8 ± 1.5 mg/dL (mean ± SEM: standard error of the mean), proteinuria was 5.3 ± 2.2 g/24 h, and the serum albumin level was 2.9 ± 0.3 g/dL. NS was seen in half of the patients with HCDD. Nasr et al. compared 64 patients with renal MIDD [15]. The mean 24-h urine protein was higher and NS occurrence was more frequent in patients with HCDD than LCDD. Oe et al. reported an overview of HCDD [24]. Extrarenal organ involvement of HCDD was observed in five cases, involving the skin, skeletal muscles, liver, and thyroid.

In our case, massive proteinuria and advanced renal failure were observed at the first visit. Organ damage was limited to the kidney. Severe hypocomplementemia was detected in our patient. The existence or nonexistence of hypocomplementemia is related to the IgG subclasses of HCDD. Soma et al. presented a first case report of γ3-HCDD in Japan and mentioned the relevance with hypocomplementemia and IgG subclasses [2]. In the previous cases, γ1 and γ3-HCDD cases showed hypocomplementemia. Particularly, severe hypocomplementemia with markedly low C3 and C4 levels were recognized in the γ3-HCDD cases. On the other hand, patients with γ2 and γ4-HCDD did not exhibit hypocomplementemia [1, 3]. Among IgG subclasses, IgG3 binds C1q most efficiently and strongly activates the classical pathway of the complement system [4]. In γ1 andγ3-HCDD, complement activation may be related enhanced C1q binding secondary to CH1 deletion. Therefore, in γ3-HCDD cases, including our patient, severely low C3 and C4 levels have been observed. In our case, immunofluorescence staining of the renal tissue revealed positivity of not only IgG, but also C3 and C1q. These findings did not contradict the classical pathway activation by IgG3. The serum complement levels correlated with the disease activity of HCDD. In previous cases, normalization of the serum complement titers with appropriate treatment was reported [5, 6].

The serum or urine monoclonal protein levels were positive in more than half of the HCDD patients (64–86% in the serum and 50–83% in the urine), but not all [24]. The absence of monoclonal protein is not sufficient to rule out HCDD. In our patient, routine serum and urine immunoelectrophoresis, serum immunofixation electrophoresis, and the serum free light chain ratio (κ /λ ratio) were examined for detection of monoclonal protein several times. However, serum and urine monoclonal protein was not found. In this case, bone marrow aspirate did not show monoclonal proliferation of plasma cells. In the previous article, a definitive diagnosis of plasma cell dyscrasia could be made in only 19% of cases of γ-HCDD [7].

Typical pathological findings of HCDD show nodular mesangial sclerosis. The mesangial nodules were generally positive for periodic acid-Schiff, trichrome blue, and silver stain. Crescentic formation is also sometimes observed [7]. Vedder et al. reported an unusual case of endocapillary proliferative glomerulonephritis due to HCDD [8]. An immunofluorescence study revealed linear positive staining on GBM and TBM for a single heavy chain with completely negative staining for light chains. In the majority of γ3-HCDD cases, complement deposition is detected. On electron microscopy, fine granular EDD along the inner aspect of the lamina densa of GBM and the outer aspect of the TBM were observed. EDD were also seen in the expanded mesangial area. Mesangial deposits tend to be more common in HCDD than LCDD [15].

The diagnosis of HCDD requires characteristic pathological findings. The following criteria were applied in previous cohort studies of MIDD; (A) positive staining for a single class of immunoglobulin (γ, α, μ) on the GBM and/or TBM, (B) completely negative staining of light chains (κ and λ), and (C) characteristic EDD observed by electron microscopy [15, 18, 24]. Renal pathological findings of this present case fulfilled these above criteria. The finding that only IgG3 is linearly positive for TBM was of particular importance. Although Aucouturier et al. suggested the hallmark of HCDD is CH1 deletion of γ-heavy chains [9], staining for CH1 is not necessarily required for a diagnosis. In addition to the pathological diagnosis, clinical features, which include NS with progressive renal failure, hypertension, and prominent hypocomplementemia, coincided with the previously reported clinical presentations.

The treatment of HCDD has not been established until now. The prognosis of HCDD is very poor historically. Kambham et al. reported 11 cases of HCDD treated with corticosteroids, cyclophosphamide, and melphalan [17]. In the 8 patients for whom follow-up data was available, 2 progressed to end-stage kidney disease, which required renal replacement therapy within less than 1 year, and 3 had worsening proteinuria and deterioration in renal function. Oe et al. reviewed the clinical outcomes and prognosis in a previous report [24]. Most HCDD patients are refractory to treatment. Within a year, 36–50% of patients develop end-stage kidney disease requiring hemodialysis. Within a mean duration of 12–76 months after onset, the mortality is about 10%. However, chemotherapy based on the regimen used for multiple myeloma (MM) was useful in some cases. Soma et al. and Oe et al. reported two cases of HCDD treated successfully using MP therapy [2, 5]. Yin et al. described a unique type of γ3-HCDD that achieved complete remission according to urinalysis by treatment with thalidomide and dexamethasone [10]. Royer et al. treated 11 young patients with MIDD by high dose chemotherapy with the support of autologous blood stem cell transplantation and reported the therapeutic benefit [20].

Recently, the utility of bortezomib has been reported. Patel et al. presented 3 patients with severe kidney manifestation of HCDD who achieved clinical remission and improvement of renal function following a bortezomib-based treatment [25]. Cohen et al. retrospectively studied 49 patients with MIDD who received intravenous bortezomib plus dexamethasone. In many patients studied, hematological and renal response was achieved. Bortezomib-based therapy is considered to be beneficial mainly in MIDD cases diagnosed earlier [26]. Bridoux et al. researched 15 patients with biopsy-proven HCDD, retrospectively. All patients received chemotherapy, including bortezomib in 10 cases. Renal clinical manifestation ameliorated in 11 patients who achieved a hematological response. In this report, the importance of early diagnosis and bortezomib-based treatment is mentioned for the preservation of renal prognosis [19]. In bortezomib-based therapy, serum free light chain response is consider to be a favorable prognostic factor for renal survival [27].

Therapeutic indicators for treating HCDD are urinalysis findings and renal function. Particularly, the serum complement level is important in γ3-HCDD patients. In our case, treatment with steroids alone had no effect at all. However, after the treatment with MP therapy, the serum albumin levels have been maintained at over 3.0 g/dL, and NS was not observed for a long period of time. The serum creatinine value was stable.

Hypocomplementemia was notably improved. For these reasons, MP therapy was considered to be beneficial in this case. Interestingly, our patient responded well to the MP therapy, although a bone marrow aspirate did not show findings of plasm cell infiltration, and monoclonal immunoglobin was not identified in the serum or urine by conventional electrophoresis and immunofixation. These results indicate that the monoclonal immunoglobin levels are very small, reflecting a strong affinity for kidney tissues. In fact, in this case, monoclonal immunoglobin was demonstrated only in a renal biopsy by immunofluorescence staining. The initiation of therapeutic intervention at an early stage of disease was indicated as a favorable prognostic factor in previous reports [2, 5, 15, 18, 24]. The response to MP therapy in this case may be interpreted by the early diagnosis. On histological findings, mild chronic changes such as tubular atrophy, interstitial fibrosis, and the absence of glomerular sclerosis lesions were also considered to be related to the treatment response.

In recent years, a review of monoclonal gammopathy of renal significance (MGRS) has been reported [28]. MGRS is a disease group of renal impairment induced by a monoclonal protein. MGRS is a new concept, and in 2012 the International Kidney and Monoclonal Gammopathy Research Group refined the definition of MGRS to distinguish these monoclonal gammopathies from monoclonal gammopathy of undetermined significance [29]. MIDD, including HCDD, is one category of MGRS. The monoclonal protein may cause kidney injury directly through non-organized deposits in MIDD. In MGRS, the amount of monoclonal protein and rapid deposition in the kidney influence the failure to detect monoclonal protein in the serum and urine. Therefore, kidney biopsy is needed for diagnosis of MGRS. In this case, the renal pathology findings were most important for the diagnosis of HCDD. Particularly, no immunofluorescence staining for light chains was a significant clue. In this case, although MM was not detected, chemotherapy based on the treatment regimen for MM was effective. More recently, it has been reported on the management of MGRS [30]. In this article, the treatment with high-dose melphalan supported by autologous peripheral blood stem cell transplantation and bortezomib-based regimens is recommended for the MIDD patients. In MGRS, early recognition and aggressive adaptation of the chemotherapeutic regimen may be crucial for renal prognosis.

## Conclusions

In conclusion, we present a case of γ3-HCDD with NS and renal dysfunction who responded to MP therapy and maintained kidney function for a long time. However, further careful observation is necessary to assess the treatment effect. In order to improve the renal outcome of HCDD, an excellent understanding of renal pathology for early diagnosis and suitable treatment methods are required. Penetration of MGRS concept and aggressive chemotherapy may improve the renal prognosis.

## Abbreviations

CKD: Chronic kidney disease
EDD: Electron-dense deposits
FITC: Fluorescein isothiocyanate
GBM: Glomerular basement membranes
HCDD: Heavy chain deposition disease
LCDD: Light chain deposition disease
LHCDD: Light and heavy chain deposition disease
MGRS: Monoclonal gammopathy of renal significance
MIDD: Monoclonal immunoglobulin deposition disease
MM: Multiple myeloma
MP: Melphalan plus prednisolone
NS: Nephrotic syndrome
PSL: Prednisolone
TBM: Tubular basement membranes
